# Personalized prediction of immunotherapy response in lung cancer patients using advanced radiomics and deep learning

**DOI:** 10.1186/s40644-024-00779-4

**Published:** 2024-09-30

**Authors:** Chien-Yi Liao, Yuh-Min Chen, Yu-Te Wu, Heng-Sheng Chao, Hwa-Yen Chiu, Ting-Wei Wang, Jyun-Ru Chen, Tsu-Hui Shiao, Chia-Feng Lu

**Affiliations:** 1 Department of Biomedical Imaging and Radiological Sciences, National Yang Ming Chiao Tung University, No. 155, Sec. 2, Linong Street, Beitou District, Taipei, 112 Taiwan; 2 Department of Chest Medicine, Taipei Veterans General Hospital, Taipei, Taiwan; 3 Institute of Biophotonics, National Yang Ming Chiao Tung University, Taipei, Taiwan; 4 School of Medicine, National Yang Ming Chiao Tung University, Taipei, Taiwan; 5 Brain Research Center, National Yang Ming Chiao Tung University, Taipei, Taiwan; 6 Department of Radiation Oncology, University of Texas Southwestern Medical Center, Dallas, TX USA

**Keywords:** Lung cancer, Outcome prediction, Deep learning, Computed tomography, Radiomics

## Abstract

**Background:**

Lung cancer (LC) is a leading cause of cancer-related mortality, and immunotherapy (IO) has shown promise in treating advanced-stage LC. However, identifying patients likely to benefit from IO and monitoring treatment response remains challenging. This study aims to develop a predictive model for progression-free survival (PFS) in LC patients with IO based on clinical features and advanced imaging biomarkers.

**Materials and methods:**

A retrospective analysis was conducted on a cohort of 206 LC patients receiving IO treatment. Pre-treatment computed tomography images were used to extract advanced imaging biomarkers, including intratumoral and peritumoral-vasculature radiomics. Clinical features, including age, gene status, hematology, and staging, were also collected. Key radiomic and clinical features for predicting IO outcomes were identified using a two-step feature selection process, including univariate Cox regression and chi-squared test, followed by sequential forward selection. The DeepSurv model was constructed to predict PFS based on clinical and radiomic features. Model performance was evaluated using the area under the time-dependent receiver operating characteristic curve (AUC) and concordance index (C-index).

**Results:**

Combining radiomics of intratumoral heterogeneity and peritumoral-vasculature with clinical features demonstrated a significant enhancement (*p* < 0.001) in predicting IO response. The proposed DeepSurv model exhibited a prediction performance with AUCs ranging from 0.76 to 0.80 and a C-index of 0.83. Furthermore, the predicted personalized PFS curves revealed a significant difference (*p* < 0.05) between patients with favorable and unfavorable prognoses.

**Conclusions:**

Integrating intratumoral and peritumoral-vasculature radiomics with clinical features enabled the development of a predictive model for PFS in LC patients with IO. The proposed model’s capability to estimate individualized PFS probability and differentiate the prognosis status held promise to facilitate personalized medicine and improve patient outcomes in LC.

**Supplementary Information:**

The online version contains supplementary material available at 10.1186/s40644-024-00779-4.

## Key points

• Immunotherapy’s unique mechanisms in lung cancer have challenged traditional tumor volume-based response assessment, requiring new prognostic factors and algorithms.

• Advanced radiomics analysis, including intratumoral imaging phenotypes and peritumoral vasculature, comprehensively quantifies the tumor characteristics and angiogenesis in lung cancer.

• Integrating clinical features, advance radiomics, and deep learning models provides a more precise prediction of the response to immunotherapy in lung cancer.

**Supplementary Information:**

The online version contains supplementary material available at 10.1186/s40644-024-00779-4.

## Introduction

Lung cancer (LC) is a prominent contributor to global cancer-related mortality. Immunotherapy (IO) has emerged as a promising therapeutic approach for LC, particularly in advanced-stage patients [1, 2]. Unlike traditional chemotherapy that directly targets and destroys cancer cells, IO harnesses the inherent capabilities of the patient’s immune system to suppress the growth of cancer cells [3]. The most widely studied immunotherapy agents for LC are immune checkpoint inhibitors (ICIs), which block inhibitory signals on T-cells [4]. ICIs have improved overall survival (OS) and quality of life in patients with LC, but not all patients benefit from these therapies [5]. Furthermore, ICIs can cause serious immune-related adverse events such as pneumonitis. Therefore, having predictive biomarkers to identify patients most likely to benefit from IO and to monitor treatment response is critical [6].

Currently, the most used biomarkers for predicting response to ICIs are programmed death ligand 1 (PD-L1) expression on tumor cells. Several clinical trials focusing on PD-L1 expression in LC patients have demonstrated that those with low PD-L1 expression may still benefit from ICI drugs. These individuals commonly receive a combination of chemotherapy and ICI therapy. Conversely, patients with high PD-L1 expression frequently exhibit response rates below 50% when treated solely with ICI monotherapy [7]. This highlights the urgent need for IO predictors in LC patients in addition to the PD-L1 expression [8]. Accurate prediction can facilitate LC management and avoid unnecessary costs and potential adverse effects of IO treatment [9, 10].

Medical imaging combined with deep-learning approaches is increasingly being used to predict the efficacy of lung cancer treatment [11]. AI methods can extract meaningful information from complex data (e.g., imaging data from radiology or histopathology images) to predict response to immunotherapy, either directly or indirectly through surrogate markers. These AI-based techniques have the potential to improve diagnostic accuracy, optimize treatment planning, predict treatment efficacy, and reduce human resource costs in the management of cancer immunotherapy [12, 13]. We expanded upon concepts from prior studies [12, 13] that leveraged diagnostic contrast CT images in developing immunotherapy predictors. This study broke new ground by integrating comprehensive clinical data, encompassing hematology, genetics, and clinical presentation, and fusing advanced imaging features with deep learning models.

Radiomics, a high-throughput method to quantify image traits based on computed tomography (CT), has shown promising results in diagnosis and prognosis for patients with LC. Intratumoral CT radiomics offers valuable insights into tumor-specific characteristics and can potentially enhance the prediction of treatment outcomes in LC patients [14]. Furthermore, tumor cells induce an immune-evasive state by releasing vascular endothelial growth factor, promoting angiogenesis, and supplying nutrients to the tumor [15, 16]. Numerous studies have demonstrated that well-structured blood vessels hinder metastasis formation and improve the delivery of chemotherapy agents to the tumor site [17, 18]. Peritumoral-vasculature radiomics can quantify subtle vascular changes and provide a comprehensive assessment of the tumor microenvironment [19]. Overall, the multi-modality vasculature characteristics offer a promising solution to enhance the management and treatment outcomes of LC patients undergoing IO.

The present study aims to develop a predictive deep-learning model based on advanced radiomics (including intratumoral and peritumor-vasculature features) and clinical features for progression-free survival (PFS) in LC patients with IO. The proposed model could provide clinicians with a non-invasive tool to identify patients most likely to benefit from IO and to monitor treatment response. The outcome of this study might help to reduce the risk of immune-related adverse events by avoiding unnecessary treatment in patients unlikely to benefit from ICIs.

## Materials and methods

### Patient cohort and image data

**Fig. 1:**
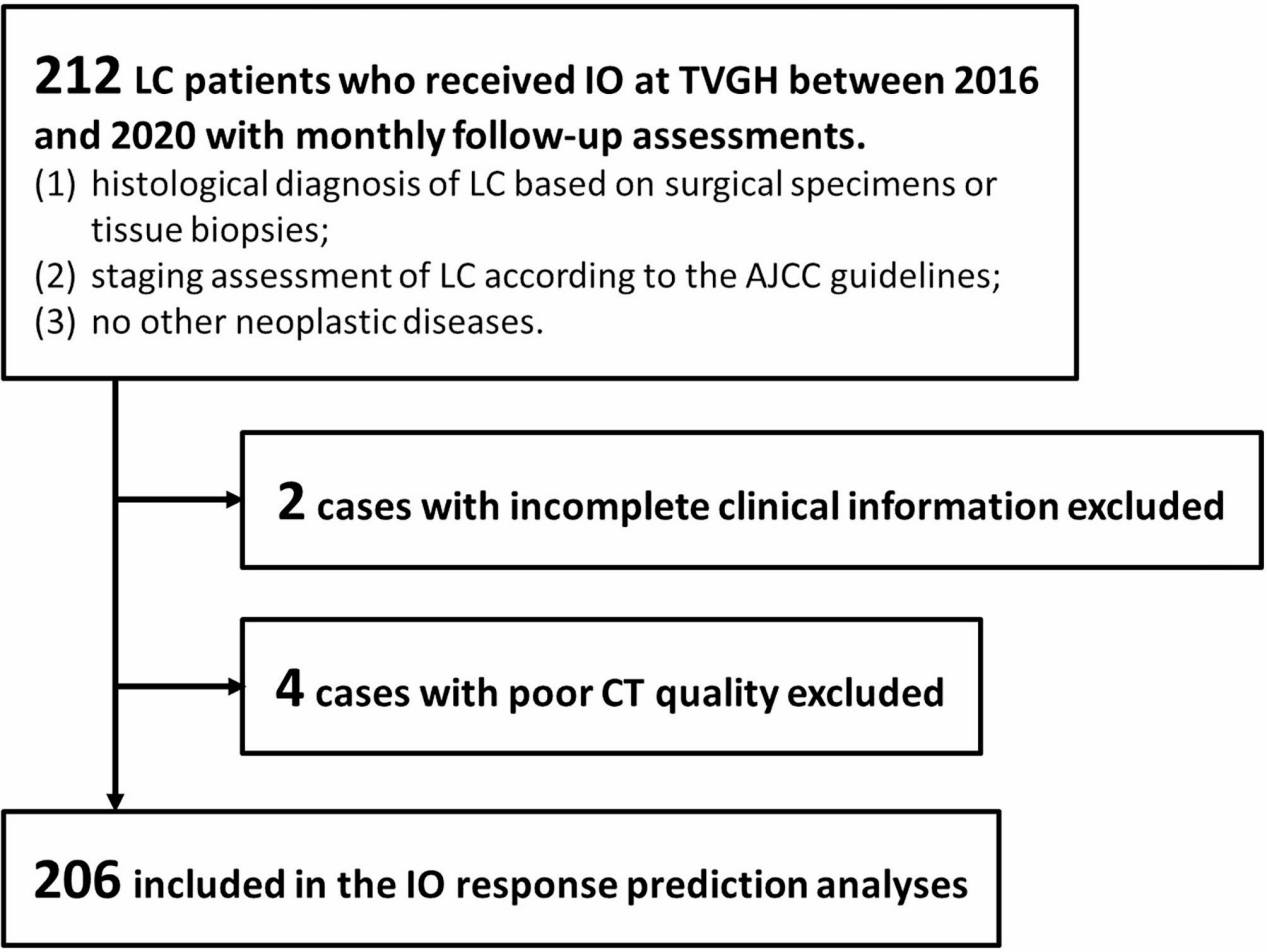
Patient flowchart for this study

The Institutional Review Board of Taipei Veterans General Hospital (TVGH) approved this retrospective study (IRB No.: 2021-09-009BCF) and waived the requirement of informed consent from patients. This study collected data from 212 LC patients who received IO treatment between 2016 and 2020 with monthly follow-up assessments. The inclusion criteria are listed as follows: (1) histological diagnosis of LC based on surgical specimens or tissue biopsies; (2) staging assessment of LC according to the American Joint Committee on Cancer (AJCC) guidelines [20] based on imaging and pathological information; (3) no other neoplastic diseases; (4) complete clinical information, such as smoking status, Eastern Cooperative Oncology Group performance status (ECOG PS) score, applied IO drugs, EGFR mutation status; and (5) adequate quality of contrast-enhanced chest CT data. Finally, 206 LC patients were included in the subsequent analyses (Fig. 1). Disease progression was identified as tumor enlargement, new metastasis, and patient death based on monthly follow-up assessments (including vital signs, blood tests, and CT scans if needed), aligning with the primary objective of our model to forecast responses to IO. Discontinuities in the follow-up procedure were documented as instances of data censoring.

Among 206 included patients, pre-treatment CT image data were acquired using several different scanners produced by five major manufacturers, including GE, Hitachi, Siemens, Toshiba, and Philips (detailed information in Supplementary Table 1). We did not limit the use of specific manufacturers or models of CT scanners to ensure that the predictive model we developed could tolerate the variability introduced by different machines. The CT scan coverage was similar, ranging from the thoracic inlet to the upper abdomen. CT images were reconstructed with a slice thickness between 1.0 and 5.0 mm. Pixel spacing ranged from 0.5 to 1.2 mm. Each slice had a matrix size of 512 × 512 pixels with a 16-bit gray-scale resolution. The peak tube voltage was 120 kVp, and the tube current was decided by automatic dose modulation.

In order to delineate the location of the tumors for the radiomics analysis, experienced radiologists and certified pulmonologists collaborated to assess the quality and delineate the region of interest (ROI) in CT scans. For ROI delineation, two specific window settings were applied to the CT images. The soft tissue window (width: 350, level: 50) aided in distinguishing tumors, collapsed lungs, and fluid components like pleural and pericardial effusions. On the other hand, the lung window (width: 1500, level: -600) was used to determine tumor borders accurately. In the cases with multiple tumors, only the primary tumor was selected as the ROI.

### Intratumoral radiomics

**Fig. 2:**
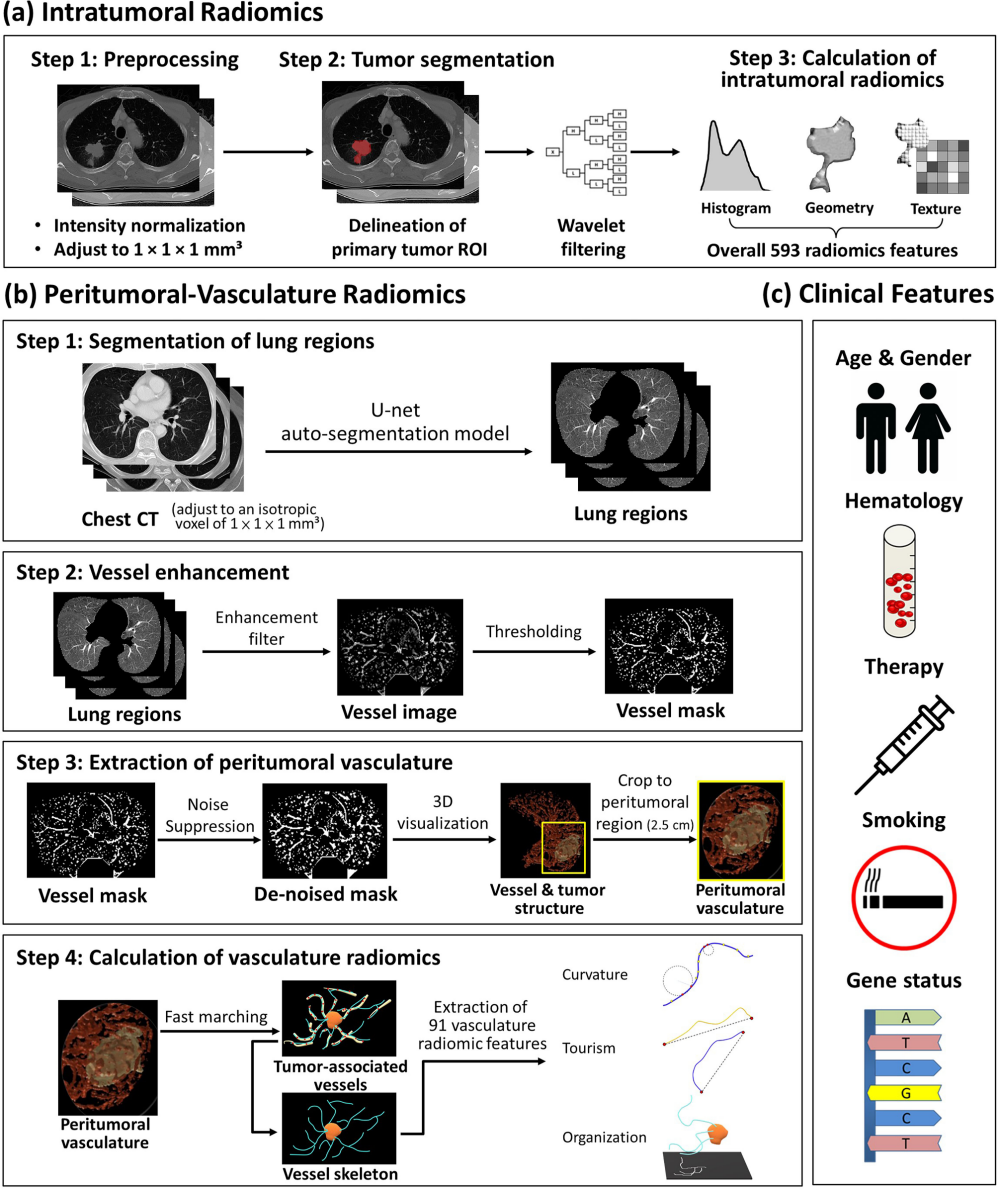
Feature processing workflow for (**a**) intratumoral radiomics, (**b**) peritumoral-vasculature radiomics, and (**c**) clinical features

We applied several preprocessing steps to the CT images, including resolution adjustment to an isotropic pixel size of 1 × 1 × 1 mm³, followed by intensity standardization using Z-score transformation to achieve standardized ranges based on the mean and standard deviation of the image data. Additionally, low-pass (L) and high-pass (H) wavelet filters were applied to the three axes of CT images, resulting in eight decomposed image sets (named LLL, LLH, LHL, LHH, HLL, HLH, HHL, and HHH images). Intratumoral radiomics analysis involved the calculation of histogram, geometry, and texture features, including gray level co-occurrence matrix (GLCM), gray level run length matrix (GLRLM), and local binary pattern (LBP), from eight wavelet-filtered images and the original CT images. The feature extraction process followed the Imaging Biomarker Standardization Initiative (IBSI) guidelines [21]. In total, 593 intratumoral radiomic features were generated for each ROI (Fig. 2a). The image preprocessing, ROI delineation, and intratumoral radiomics analysis were performed using the Multimodal Radiomics Platform, a MATLAB-based graphic user interface (available online: http://cflu.lab.nycu.edu.tw/MRP_MLinglioma.html, accessed on 9 May 2024) [22, 23]. Supplementary Table S2 provides the formulas used for intratumoral radiomics analysis.

### Peritumoral-vasculature radiomics

After the adjustment of image resolution to an isotropic voxel of 1 × 1 × 1 mm³, a pre-trained U-Net model based on 231 routine CT cases (acquired from different scanner manufacturers) was employed to automatically segment the bilateral lung areas on CT images [24]. Afterward, radiomic features of tumor-associated vasculature were extracted through a stepwise process. First, a multi-scale 3D vessel enhancement filter was utilized to enhance vessel-like structures [25]. Afterward, Otsu’s thresholding method was applied to the vessel enhancement image to extract voxels associated with the vasculature. Morphological operations were subsequently applied to eliminate noise and non-vessel artifacts. Finally, a cubic region, covering a 2.5-cm extension from the tumor volume along three directions, was extracted. A fast-marching algorithm was employed on the segmented vasculature to identify the center lines of vessels and partition the vessel network into distinct branches [26]. Ninety-one vasculature radiomic features were computed, comprising 61 quantitative vessel morphology features (e.g., curvature and torsion) and 30 features describing the organization of the peritumoral vasculature (Fig. 2b). The calculation of vasculature radiomic features is provided in Supplementary Table S3. The proportion of outliers (values located outside the three standard deviations from the mean value) was computed for each normalized radiomic feature, and features with outlier proportions exceeding 20% of the entire dataset were then removed from consideration. The peritumoral-vasculature radiomics analysis was also performed using the Multimodal Radiomics Platform (available online: http://cflu.lab.nycu.edu.tw/MRP_MLinglioma.html, accessed on 9 May 2024) with QuanTAV toolbox (available online: https://github.com/ccipd/QuanTAV, accessed on 9 May 2024).

### Prediction models and statistics

**Fig. 3:**
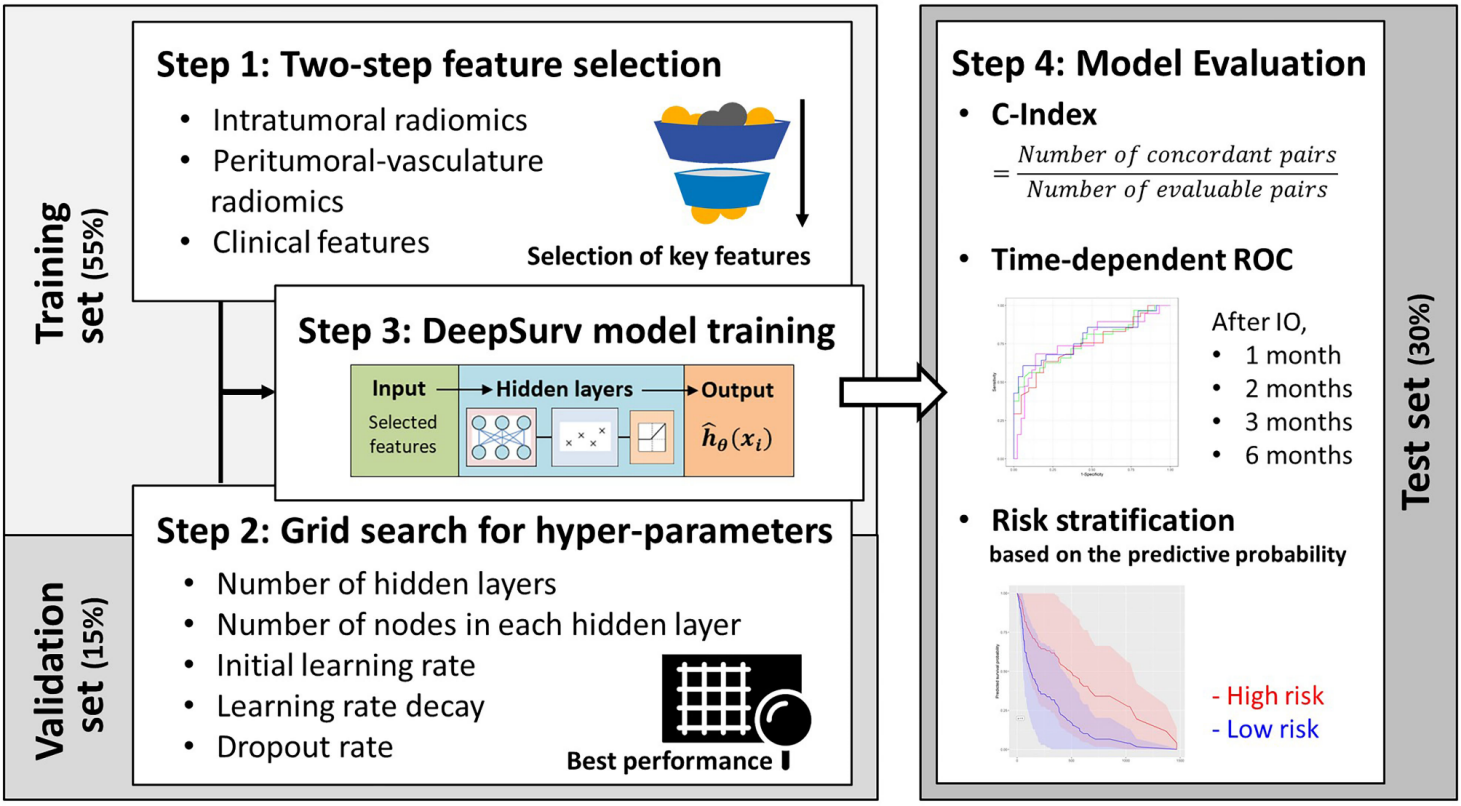
The workflow of proposed prediction models for immunotherapy response

In this study, the patient data were first divided into training (55% of cases), validation (15%), and testing (remaining 30%) sets using simple random sampling. Figure 3 shows the workflow to construct the prediction models for the IO response. To identify key radiomic and clinical features for predicting IO outcomes, a two-step feature selection was applied to the training dataset. In the first step, univariate Cox proportional regression for radiomic features and the chi-squared test for clinical features were used to quickly eliminate a large number of redundant features [27, 28], with only those having *p* < 0.05 advancing to the second step. The second step involved the implementation of a sequential forward selection (SFS) [29] algorithm to further consider feature interactions and eliminate highly correlated features.

DeepSurv, a deep learning model built on the Cox proportional hazards (CPH) framework, was employed to predict PFS after IO treatment. This model employed a multilayer perceptron network to embed nonlinear properties into the hazard function, improving the survival prediction [30, 31]. In this study, we used selected radiomic and clinical features as inputs of DeepSurv models. The model output, the estimated hazard function, was further utilized to predict personalized PFS curves for each patient. The architecture and parameter configuration of the proposed DeepSurv model are depicted in Supplementary Figure S1.

Three DeepSurv models were constructed, incorporating different sets of features: (1) clinical features only (e.g., age, sex, hematology, molecular status, metastatic information, and histopathology); (2) clinical and intratumoral radiomic features; and (3) clinical features, intratumoral radiomics, and peritumoral-vasculature radiomics. We further leveraged Shapley Additive Explanations (SHAP) to assess the contribution of individual feature to outcome prediction in DeepSurv models. This process involved sorting and visualizing the importance of each feature in shaping the model’s outcomes [32]. In this study, the hyper-parameters were determined through the grid search method [33]. The selection of hyper-parameters was mainly based on the prediction performance, i.e., concordance index (C-index), in the validation set,. Only when multiple models (with different combinations of hyper-parameters) presented the same C-index value would we select the one with the shortest training time. Supplementary Table S4 presents the selected hyper-parameters based on the grid search.

Patients will be stratified into high-risk and low-risk groups based on the PFS probability predicted by the DeepSurv model at the median PFS time point (3.3 months). Patients with a predicted PFS probability higher than 50% at 3.3 months will be classified as the low-risk group, while those with a predicted PFS probability of 50% or lower will be classified as the high-risk group. A log-rank test was employed to evaluate the statistical differences in Kaplan-Meier (KM) survival curves between the high-risk and low-risk groups. The DeepSurv models were also used to predict progression status at four follow-up time points (i.e., 1, 2, 3, and 6 months after the IO treatment). The performance of DeepSurv models, including time-dependent receiver operating characteristic (ROC) curves, area under the ROC curve (AUC), C-index, sensitivity, and specificity, was evaluated using the testing dataset (remaining 30% of patients). To calculate the C-index, consider all possible pairs of subjects in the dataset. For each pair, determine if the subject with the shorter actual survival time also has a shorter predicted survival time. The C-index is the ratio of concordant pairs to the total number of evaluable pairs [34]:

$$\:C-index=\frac{Number\:of\:concordant\:pairs}{Total\:number\:of\:evaluable\:pairs}$$

To compare performance among three DeepSurv models, we utilized a bootstrap random sampling technique 100 times, followed by the paired t-test of AUC values. Feature selection and training of the DeepSurv models were conducted using the R DeepSurv package (available online: https://rdrr.io/cran/survivalmodels/src/R/deepsurv.R, accessed on 6 May 2023).

## Results

### Characteristics of enrolled patients

Over 50% of the recruited LC patients were at stage IVB with a median PFS of 3.3 months after the IO treatment. Smokers accounted for more than 55% of the patient cohort. Adenocarcinoma was the predominant histological type (71.3%), and most patients had a wild-type epidermal growth factor receptor (61.1%). Adverse drug reactions among the patients were 18.4%. Detailed clinical characteristics of the 206 recruited NSCLC patients are summarized in Table 1.

**Table 1:** Characteristics of 206 recruited LC patients receiving immunotherapy

Characteristics	Value
**Age**, **median (IQR)**	62.2 (12.8)
**Progression-free survival**, **median (range)**	3.3(0.1–48.9)
**Gender**	
Female, *N* (%)	65 (31.6)
**Smoking status**	
Smoker, *N* (%)	117 (56.8)
**ECOG PS score**	
0, *N* (%)	81 (39.3)
1, *N* (%)	124 (60.2)
2, *N* (%)	1 (0.5)
**Tumor stage before immunotherapy**	
Stage II, *N* (%)	1 (0.5)
Stage III, *N* (%)	5 (2.4)
Stage IVA, *N* (%)	79 (38.4)
Stage IVB, *N* (%)	121 (58.7)
**Applied immunotherapy drug**	
Nivolumab, *N* (%)	84 (40.8)
Pembrolizumab, *N* (%)	71 (30.4)
Atezolizumab, *N* (%)	40 (19.4)
Durvalumab, *N* (%)	9 (4.4)
Avelumab, *N* (%)	2 (1.0)
**Histology**	
Adenocarcinoma, *N* (%)	147 (71.3)
Squamous cell carcinoma, *N* (%)	27 (13.1)
Not otherwise specified carcinoma, *N* (%)	13 (6.3)
Lymphoepithelial-like carcinoma, *N* (%)	1 (0.5)
Sarcomatoid carcinoma, *N* (%)	1 (0.5)
Large cell carcinoma, *N* (%)	1 (0.5)
Squamous cell + Adenocarcinoma, *N* (%)	1 (0.5)
Small cell lung cancer, *N* (%)	15 (7.3)
**EGFR mutation status**	
Wild type, *N* (%)	126 (61.1)
Exon 19 deletion, *N* (%)	17 (8.3)
Exon 20 deletion, *N* (%)	3 (1.5)
Exon 21 L858R substitution, *N* (%)	13 (6.3)
Other, *N* (%)	47 (22.8)
**Tumor Proportion Score (PD-L1)**	
High expression, *N* (%)	41 (20.0)
Normal expression, *N* (%)	31 (15.0)
No expression, *N* (%)	38 (18.4)
Not available, *N* (%)	96 (46.6)
**Treatment prior to the first immunotherapy**	
Chemotherapy, *N* (%)	188 (91.2)
Radiotherapy, *N* (%)	117 (56.8)
Lung surgery, *N* (%)	37 (18.0)
**Other Treatment Combined with immunotherapy**	
Chemotherapy, *N* (%)	75 (36.4)
Target therapy, *N* (%)	4 (1.9)
**Immunotherapy as first-line treatment**	
Yes, *N* (%)	10 (4.9)
No, *N* (%)	196 (95.1)
**Brain metastasis**	
Never, *N* (%)	126 (61.2)
Initial diagnosis, *N* (%)	50 (24.3)
After initial diagnosis, *N* (%)	30 (14.5)
**Pneumonitis**	
Yes, *N* (%)	15 (7.3)
**Adverse event**	
Yes, *N* (%)	38 (18.4)
**Total proteins**	
High, *N* (%)	202 (98.1)
Normal, *N* (%)	4 (1.9)
**Mean corpuscular volume**	
High, *N* (%)	7 (3.4)
Normal, *N* (%)	188 (91.3)
Low, *N* (%)	11 (5.3)

Definitions: total proteins: high: > 6 g/dl, low: < 6 g/dl; mean corpuscular volume: high > 100 fl., normal:80–100 fl., low < 80 fl

Abbreviations: ECOG Eastern Cooperative Oncology Group, EGFR Epidermal growth factor receptor, PS Performance status

### Key predictors for prognosis after the IO treatment

A feature selection procedure was employed to identify discriminative features for predicting tumor response. The selected features were utilized to train DeepSurv models. Supplementary Table S5 presents the list of features included in the PFS prediction, along with corresponding hazard ratios, to show the potential influence on the prediction. Visualization results of the SHAP model have been included in Supplementary Figure S2 for additional insight and reference, facilitating further examination of feature contributions to the model. Tumor volume, total protein, and mean corpuscular volume were associated with the PFS. A poorer prognosis was associated with a larger intratumoral local heterogeneity and tumor diameter. Additionally, increased mean torsion on vessel branches surrounding the tumor and overall curvature were identified as features closely associated with poor response to immunotherapy in LC patients.

### Prediction performance of DeepSurv models

**Fig. 4:**
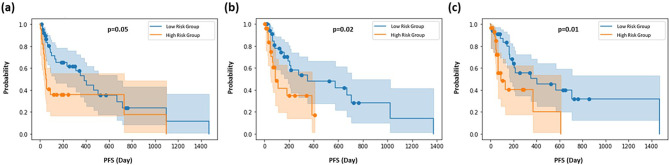
Distribution of KM survival curves predicted by DeepSurv models. The figure displays the KM survival curves for patients in the testing set based on (**a**) clinical features, (**b**) clinical + intratumoral radiomics, and (**c**) clinical + intratumoral + peritumoral-vasculature radiomics

The distribution of KM curves predicted by DeepSurv models is depicted in Fig. 4. These curves were generated based on three different DeepSurv models with different input features: **Model (1)** clinical features, **Model (2)** clinical features and intratumoral radiomics, and **Model (3)** clinical features, intratumoral radiomics, and peritumoral-vasculature radiomics. **Model 2** and **Model 3** exhibited significant differences (*p* < 0.05) in the PFS curves between the high-risk and low-risk groups.

**Fig. 5:**
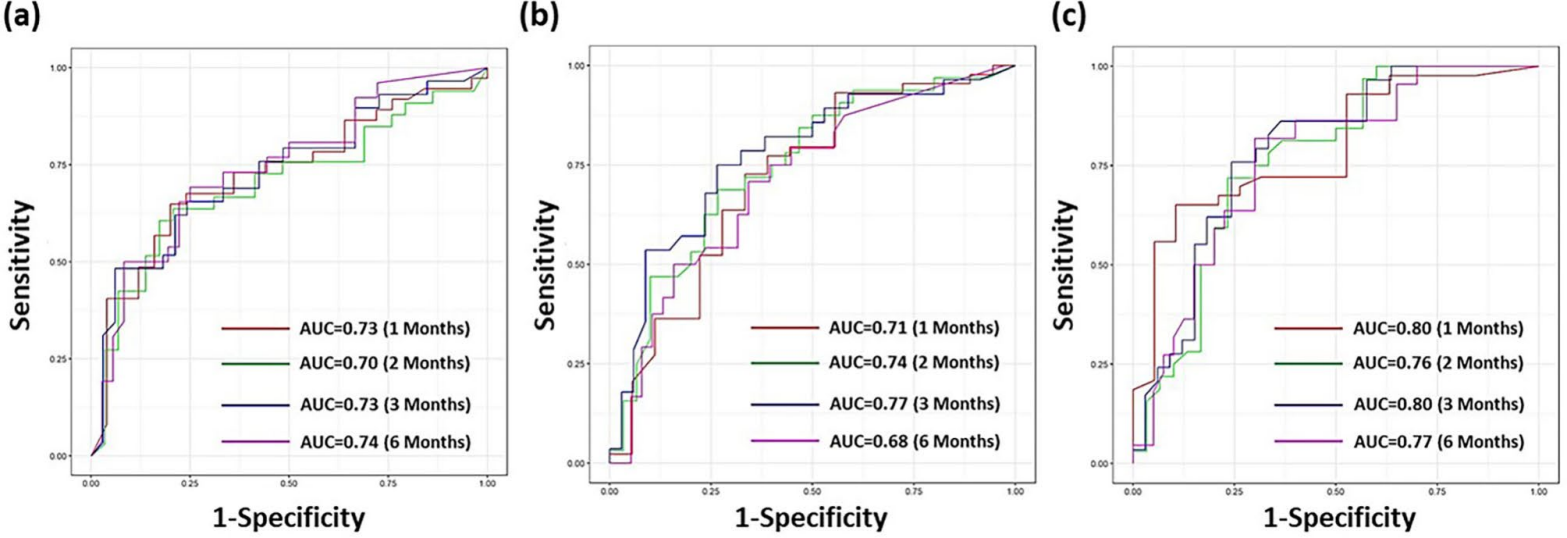
Time-dependent prediction results for progression-free survival (PFS) after immunotherapy. ROC curves of DeepSurv models based on (**a**) Clinical Features, (**b**) Clinical + Intratumoralal Radiomics, and (**c**) Clinical + Intratumoralal + Peritumoral Radiomics

We further evaluated the prediction performance of DeepSurv models using the testing dataset at four critical clinical follow-up time points (1, 2, 3, and 6 months). The time-dependent ROC curves displayed in Fig. 5 illustrate the performance of each prediction model. The clinical-based model showed AUCs ranging from 0.68 to 0.77, with a C-index of 0.66. The clinical and intratumoral radiomics-based model demonstrated AUCs between 0.70 and 0.74, with a C-index of 0.68. The model combining clinical, intratumoral, and peritumoral-vasculature radiomics achieved AUCs ranging from 0.76 to 0.80, with a C-index of 0.83. Statistical comparisons of prediction performance between three DeepSurv models are summarized in Table 2. Overall, the DeepSurv model incorporating clinical, intratumoral, and peritumoral-vasculature radiomics significantly outperformed (*p* < 0.001 in AUCs) two other models for predicting progression risk at each time point.

**Table 2:** Prediction performance and statistical comparisons of PFS among three models

Estimate	Prediction time points			
	1 month	2 months	3 months	6 months
**Model1: Clinical features (C-index = 0.66)**				
AUC (95% CI ^a^)	0.73 (0.73–0.93)	0.70 (0.64–0.82)	0.73 (0.65–0.86)	0.74 (0.66–0.89)
Sensitivity	68%	67%	69%	69%
Specificity	68%	69%	67%	69%
**Model2: Clinical + intratumoral (C-index = 0.68)**				
AUC (95% CI)	0.71 (0.60–0.75)	0.74 (0.64–0.79)	0.77 (0.68–0.82)	0.68 (0.67–0.70)
Sensitivity	73%	72%	71%	71%
Specificity	67%	67%	74%	66%
**Model3: Clinical + intratumoral + peritumoral-vasculature (C-index = 0.83)**				
AUC (95% CI)	0.80 (0.71–0.93)	0.76 (0.72–0.84)	0.80 (0.71–0.88)	0.77 (0.70–0.95)
Sensitivity	70%	75%	76%	82%
Specificity	74%	70%	76%	70%
**Statistical comparison in AUC (** ***p*** **-value)**				
Model 1 vs. 3	< 0.001	< 0.001	< 0.001	< 0.001
Model 2 vs. 3	< 0.001	0.004	< 0.001	< 0.001

^a^ CI: Confidence interval in 100 times bootstrap sampling

### Added values to personalized outcome prediction using advanced radiomics

**Fig. 6:**
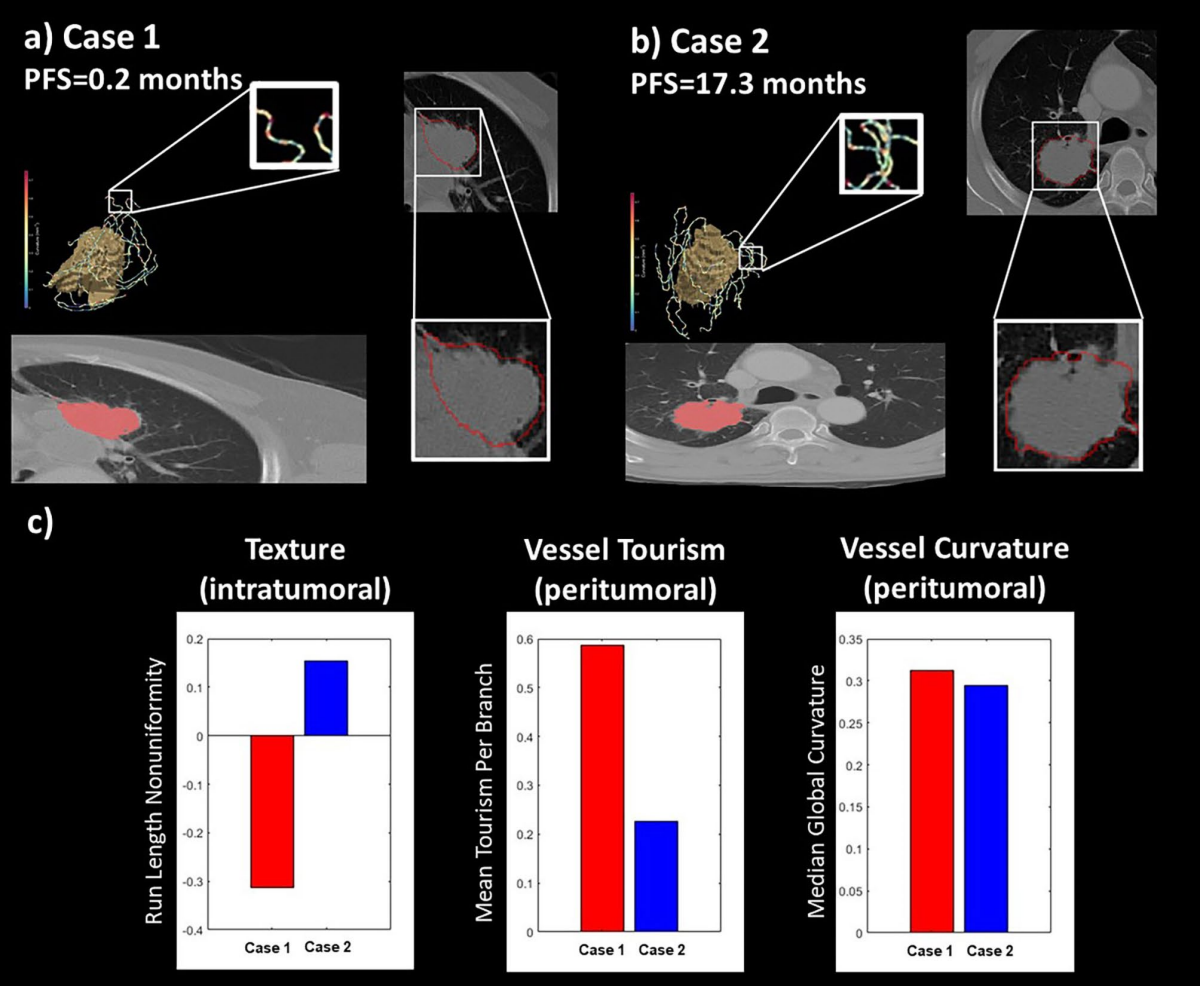
Representative cases for the IO outcome prediction. The outcome of IO treatment is associated with the characteristics of the peritumoral vasculature and the physical properties of the tumor as observed on CT images. In two cases with similar tumor size, Case 1 (**a**), with a poorer prognosis, exhibited higher local heterogeneity of the tumor and curvature and tortuosity of the peritumoral vessels compared to Case 2 (**b**), with a better prognosis. Case 1 (a) also showed growth primarily in the area near the chest wall, with limited distribution of blood vessels around the tumor. These findings suggest that the effects of IO treatment and drug delivery may be influenced by these factors

Figure 6 presents the results of intratumoral and peritumoral vascular radiomics in predicting IO. A case with poor IO results (PFS = 0.2 months, Fig. 6a) compared to a good IO result (PFS = 17.3 months, Fig. 6b) showed a higher skewness per branch of peritumoral-vasculature and local heterogeneity within the tumor (Fig. 6c). These vascular characteristics and tumor heterogeneity resulted in poor treatment outcomes. Furthermore, the tumor located near the thoracic wall, as shown in Fig. 6a, presents a poor prognosis.

## Discussion

The expression of PD-L1 in tumor cells is widely studied and has been approved as a well-known predictor of IO treatment in LC patients. Despite the predictive effect of the PD-L1 biomarker, there are still several limitations to its practical use, including variation over time and tumor locations [35], inconsistent prediction efficacy [36, 37], and unclear interactions with EGFR and ALK mutations [38]. Accordingly, we aimed to identify more reliable predictors of IO outcome to improve clinical management of patients with advanced LC.

Several studies have indicated that utilizing radiomics and machine learning technologies may benefit LC patients with IO [18, 39, 40]. One previous study proposed that analyzing textural changes in CT scans before and after treatment could provide valuable insights into the tumor microenvironment’s evolution, leading to enhanced prediction of IO response [40]. Another study utilized vessel tortuosity-related features and reported an AUC of 0.67 for predicting treatment outcomes of immune checkpoint inhibitors [41]. Although these findings highlighted the potential of radiomics in IO response prediction, the performance in predicting PFS and OS could only achieve a C-index range of 0.61 to 0.64. Our study showed promising results for the PFS prediction at four follow-up time points (AUC = 0.76 to 0.80) with a C-index of 0.83 by integrating intratumoral radiomics, peritumoral-vasculature radiomics, and clinical features with deep-learning model.

We highlighted significance of tumor heterogeneity and characteristics of the peritumoral-vasculature in predicting PFS in LC patients. Patients with worse PFS tended to exhibit increased tumor volumes and global intratumoral heterogeneity while demonstrating reduced local heterogeneity (Supplementary Table S5). This finding was associated with tumor histopathology, where volumetric and textural features derived from CT images might indicate tumor hypoxia status [42]. Larger tumors were more susceptible to hypoxia and exhibited greater global heterogeneity on CT images, which aligned with tumor survival and treatment resistance [43]. Previous studies suggested that the tumor volume was a potential prognostic indicator for the advanced LC [44, 45]. However, in several cases with similar tumor sizes, different outcomes were observed in our patient cohort. Our findings revealed that elevated mean torsion and overall curvature of the peritumoral-vascular branches were strongly associated with the emergence of IO resistance in advanced LC patients, consistent with prior research [41].

Our results revealed a significant correlation (*p* < 0.05) between the laboratory blood analysis and PFS following IO in LC patients. High mean corpuscular volume and low total protein levels indicated unfavorable PFS outcomes after IO. Previous studies have reported that elevated mean corpuscular volume of red blood cell is associated with potential folate deficiency, which can disrupt crucial processes such as DNA methylation, replication, synthesis, and repair [46, 47]. Conversely, the decline in total serum protein may lead to pronounced constitutional symptoms and compromise tolerance to intensive treatment [48]. These findings underscored the clinical relevance of these blood test indices as prognostic markers in LC patients. Future investigations integrating vessel-based imaging and laboratory biomarkers may enhance patient stratification and outcome.

While integrating advanced radiomics, hematology, and clinical data can offer a more comprehensive prognostic assessment for patients receiving IO, selecting key features and handling the interaction among features was critical to avoid the risk of overfitting and improve the model performance. The proposed DeepSurv model, which is designed by combining multi-layer perceptron architecture with Cox proportional hazards framework, gained considerable attention for survival prediction [31]. The personalized PFS curves generated by the DeepSurv model provided valuable insights into the likelihood of tumor progression following IO. Since tumor progression can occur at various time points during follow-up, using time-dependent ROC curves became crucial for assessing progression status at critical time intervals, especially in advanced LC cohorts. Most patients included in this study had advanced stage IV LC, with a median PFS of approximately three months. Early personalized survival predictions can aid in subsequent patient management.

This study revealed the personalized prognostic benefit of advanced radiomics and deep-learning models in LC patients treated with IO. However, several limitations of this study should be noted. Firstly, it was a retrospective study based on data from a single hospital, which might limit the generalizability of the findings to a broader population. To establish the reliability and robustness of the radiomics and deep learning risk assessment models, further validation using independent and prospective datasets from multiple institutions is required. Secondly, the PD-L1 testing did not show a predictive effect on the response to IO treatment. This could be attributed to the lack of PD-L1 testing results in nearly half of the patients. The missing data might reduce the predictive capability of PD-L1 expression for IO outcome. To address this limitation, prospective data collection is needed to assess the predictive value of PD-L1 expression for IO treatment response. Furthermore, most patients in this cohort received ICIs after chemotherapy or surgery, as IO was not widely used in the first-line treatment of advanced lung cancer. Consequently, the model performance should be further evaluated where IO is employed as the primary treatment. The presence of mortality as a potential competing risk factor in PFS analysis could influence the interpretability of our findings. Moreover, it should be noted that the stability and robustness of DeepSurv models might benefit from including a larger dataset with more comprehensive patient follow-up data. Finally, establishing standardized protocols, including image processing and feature calculation, for peritumoral-vasculature radiomics analysis is essential to enhance the model robustness and reliability.

## Conclusions

In conclusion, IO has revolutionized the treatment of LC by targeting immune checkpoints to improve patient outcomes. This study reported the potential of intratumoral and peritumoral-vasculature radiomics with DeepSurv models to predict IO response. Our findings provided insights into tumor heterogeneity, tumor-associated vasculature, and blood analysis as prognostic markers for personalized treatment strategies in advanced LC.

## Electronic supplementary material

Below is the link to the electronic supplementary material.

Supplementary Material 1

## Data Availability

Data is provided within the manuscript or supplementary information files. The raw data cannot be publicly available for ethical and legal reasons. However, researchers can submit inquiries for analyzed data to the corresponding authors upon reasonable request.

## Abbreviations

LC: Lung cancer
IO: Immunotherapy
ICI: Immune checkpoint inhibitor
OS: Overall survival
PD-L1: Programmed death ligand 1
CT: Computed tomography
PFS: Progression-free survival
AJCC: American Joint Committee on Cancer
ROI: Region of interest
GLCM: Gray-level co-occurrence matrix
GLRLM: Gray level run length matrix
LBP: Local binary pattern
IBSI: Imaging Biomarker Standardization Initiative
SFS: Sequential forward selection
SHAP: SHapley Additive exPlanations
CPH: Cox proportional hazards
KM: Kaplan-Meier
ROC: Receiver operating characteristic
AUC: Area under the curve
C-index: Concordance index
EGFR: Epidermal growth factor receptor
ALK: Anaplastic lymphoma kinase
