# Supplementary material for: Personalized prediction of immunotherapy response in lung cancer patients using advanced radiomics and deep learning
Source: Cancer Imaging. 2024 Sep 30;24:129. doi: 10.1186/s40644-024-00779-4 (PMC11440728; doi:10.1186/s40644-024-00779-4)
Supplement: Supplementary file 1 — Supplementary Material 1 [file 40644_2024_779_MOESM1_ESM.pdf]

SUPPLEMENTAL MATERIALS

for

Personalized Prediction of Immunotherapy Response in Lung Cancer Patients Using Advanced Radiomics and Deep Learning

by

Chien-Yi Liao, Yuh-Min Chen, Yu-Te Wu, Heng-Sheng Chao, Hwa-Yen Chiu, Ting-Wei Wang, Jyun-Ru Chen, Tsu-Hui Shiao, Chia-Feng Lu

Supplementary Table S1. CT Scanner and imaging parameters for the image acquisition in 206 Patients

Manufacturer-Model Name	Patient Number	Pixel Spacing (mm)	Matrix Size	Slice Thickness (mm)	Tube Voltage (kVp)	Tube Current (mA)
GE MEDICAL SYSTEMS-BrightSpeed	3	0.7-0.8	512×512	1.25-5.0	120	160-347
GE MEDICAL SYSTEMS-Discovery STE	1	1.0	512×512	3.75	120	32
GE MEDICAL SYSTEMS-LightSpeed VCT	15	0.6-0.9	512×512	1.25-5.0	120	100-407
Hitachi Medical Corporation-SCENARIA	1	0.7	512×512	2.5	120	211
SIEMENS-Emotion 16	3	0.6-0.8	512×512	5.0	130	100-266
SIEMENS-Sensation 16	28	0.5-0.8	512×512	5.0	120	107-280
SIEMENS-SOMATOM Definition Flash	25	0.6-0.9	512×512	5.0	120	294-1566
TOSHIBA-Aquilion	32	0.5-0.9	512×512	2.0-5.0	120	109-359
TOSHIBA-Aquilion ONE	2	0.5-0.6	512×512	2.0	120	40-50
TOSHIBA-Aquilion PRIME	15	0.6-0.8	512×512	1.0-5.0	120	114-363
TOSHIBA-Aquilion PRIME SP	2	0.6-0.7	512×512	5.0	120	156-275
Philips-Brilliance 64	18	0.6-0.9	512×512	5.0	120	139-497
Philips-Brilliance Big Bore	2	1.2	512×512	5.0	120	60
Philips-iCT 256	59	0.6-0.9	512×512	5.0	120	172-670

Supplementary Table S2. The formulae for the calculation of primary radiomic features.

**Intensity-based features (first-order statistics)**<br>**X** denotes the intensity vector with *N* voxels of the tumor ROIs; $\bar{X}$, the mean of **X**; **P**, the first-order histogram with N_l discrete intensity levels.			
**Feature**	**Formula**	**Feature**	**Formula**
1. Energy	$\sum_{i=1}^{N}\mathbf{X}(i)^2$	2. Entropy	$\sum_{i=1}^{N_l}\mathbf{P}(i)log_2\mathbf{P}(i)$
3. Kurtosis	$\frac{\frac{1}{N}\sum_{i=1}^{N}(\mathbf{X}(i)-\bar{X})^4}{\left(\sqrt{\frac{1}{N}\sum_{i=1}^{N}(\mathbf{X}(i)-\bar{X})^2}\right)^2}-3$	4. Maximum	$\max(\mathbf{X})$
5. Mean	$\frac{1}{N}\sum_{i=1}^{N}\mathbf{X}(i)$	6. Mean absolute deviation	$\frac{1}{N}\sum_{i=1}^{N}\mathrm{abs}(\mathbf{X}(i)-\bar{X})$
7. Median	$\mathrm{median}(\mathbf{X})$	8. First quartile	Value that splits off the lowest 25% of data from the highest 75%
9. Third quartile	Value that splits off the highest 25% of data from the lowest 75%	10. Minimum	$\min(\mathbf{X})$
11. Range	$\max(\mathbf{X})-\min(\mathbf{X})$	12. Root mean square (RMS)	$\sqrt{\frac{\sum_{i=1}^{N}\mathbf{X}(i)^2}{N}}$
13. Skewness	$\frac{\frac{1}{N}\sum_{i=1}^{N}(\mathbf{X}(i)-\bar{X})^3}{\left(\sqrt{\frac{1}{N}\sum_{i=1}^{N}(\mathbf{X}(i)-\bar{X})^2}\right)^3}$	14. Standard deviation	$\sqrt{\frac{1}{N}\sum_{i=1}^{N}(\mathbf{X}(i)-\bar{X})^2}$
15. Uniformity	$\sum_{i=1}^{N_l}\mathbf{P}(i)^2$	16. Variance	$\frac{1}{N}\sum_{i=1}^{N}(\mathbf{X}(i)-\bar{X})^2$
**Shape- and Size-based features**<br>*V*, tumor volume; *A*, surface area of the volume			
17. Compactness 1	$\frac{V}{\sqrt{\pi}A^{3/2}}$	18. Compactness 2	$36\pi\frac{V^2}{A^3}$
19. Maximum 3D diameter	The largest pairwise Euclidean distance between voxels on the surface of the tumor volume.	20. Spherical disproportion	$\frac{A}{4\pi R^2}$
21. Sphericity	$\frac{\pi^{\frac{1}{3}}(6V)^{\frac{2}{3}}}{A}$	22. Surface area	$A=\sum_{i=1}^{Ns}\frac{1}{2}\lvert a_ib_i\times a_ic_i\rvert$<br>*Ns*, total number of triangles covering the surface; *a*, *b*, and *c*, triangle vertices
23. Surface to volume ratio	$\frac{A}{V}$	24. Volume	Number of pixels in the tumor region multiplied by the voxel size
**Textural features (gray-level co-occurrence matrix based features)**<br>$\mathbf{P}(\delta,\alpha)$, co-occurrence matrix for an arbitrary distance δ and direction α; N_g , number of discrete intensity levels in the image; p_x(i), marginal row probabilities; p_y(i), marginal column probabilities; μ_x, mean of p_x; μ_y, mean of p_y; σ_x, standard deviation of p_x; σ_y, standard deviation of p_y; *HXY*, entropy of **P**; *HX*, entropy of p_x; *HY* , entropy of p_y;<br>$p_{x+y}(k)=\sum_{i=1}^{N_g}\sum_{j=1}^{N_g}\mathbf{P}(i,j)\,,i+j=k,k=2,3,\ldots,2N_g$;<br>$p_{x-y}(k)=\sum_{i=1}^{N_g}\sum_{j=1}^{N_g}\mathbf{P}(i,j)\,,\lvert i-j\rvert=k,k=0,1,\ldots,N_g-1$;<br>$\mathrm{HX}=-\sum_{i=1}^{N_g}p_x(i)\log_2(p_x(i)),\ \mathrm{HY}=-\sum_{i=1}^{N_g}p_y(i)\log_2\left(p_y(i)\right)$;<br>$\mathrm{HXY1}=-\sum_{i=1}^{N_g}\sum_{j=1}^{N_g}\mathbf{P}(i,j)\log_2(p_x(i)p_y(j))\ ,\ \mathrm{HXY2}=-\sum_{i=1}^{N_g}\sum_{j=1}^{N_g}p_x(i)p_y(j)\log_2(p_x(i)p_y(j))$			
25. Autocorrelation	$\sum_{i=1}^{N_g}\sum_{j=1}^{N_g}ij\mathbf{P}(i,j)$	26. Cluster Prominence	$\sum_{i=1}^{N_g}\sum_{j=1}^{N_g}[\boldsymbol{i}+\boldsymbol{j}-\boldsymbol{\mu_x}-$

			$\boldsymbol{\mu_y}]^4 \mathbf{P}(i,j)$
27. Cluster Shade	$\sum_{i=1}^{N_g}\sum_{j=1}^{N_g}[\boldsymbol{i}+\boldsymbol{j}-\boldsymbol{\mu_x}-\boldsymbol{\mu_y}]^3 \mathbf{P}(i,j)$	28. Cluster Tendency	$\sum_{i=1}^{N_g}\sum_{j=1}^{N_g}[\boldsymbol{i}+\boldsymbol{j}-\boldsymbol{\mu_x}-\boldsymbol{\mu_y}]^2 \mathbf{P}(i,j)$
29. Contrast	$\sum_{i=1}^{N_g}\sum_{j=1}^{N_g}\lvert\boldsymbol{i}-\boldsymbol{j}\rvert^2 \mathbf{P}(i,j)$	30. Correlation	$\sum_{i=1}^{N_g}\sum_{j=1}^{N_g}\frac{ij\mathbf{P}(i,j)-\mu_x(i)\mu_y(j)}{\sigma_x(i)\sigma_y(j)}$
31. Difference entropy	$\sum_{i=0}^{N_g-1} p_{x-y}(i)log_2[p_{x-y}(i)]$	32. Dissimilarity	$\sum_{i=1}^{N_g}\sum_{j=1}^{N_g}\lvert\boldsymbol{i}-\boldsymbol{j}\rvert\mathbf{P}(i,j)$
33. Energy	$\sum_{i=1}^{N_g}\sum_{j=1}^{N_g}[\mathbf{P}(i,j)]^2$	34. Entropy (*HXY*)	$-\sum_{i=1}^{N_g}\sum_{j=1}^{N_g}\mathbf{P}(i,j)\log_2(\mathbf{P}(i,j))$
35. Homogeneity 1	$\sum_{i=1}^{N_g}\sum_{j=1}^{N_g}\frac{\mathbf{P}(i,j)}{1+\lvert i-j\rvert}$	36. Homogeneity 2	$\sum_{i=1}^{N_g}\sum_{j=1}^{N_g}\frac{\mathbf{P}(i,j)}{1+\lvert i-j\rvert^2}$
37. Informational measure of correlation 1	$\frac{HXY-HXY1}{\max(HX,HY)}$	38. Informational measure of correlation 2	$\sqrt{1-e^{-2(HXY2-HXY)}}$
39. Inverse Difference Moment Normalized	$\sum_{i=1}^{N_g}\sum_{j=1}^{N_g}\frac{\mathbf{P}(i,j)}{1+\left(\frac{\lvert i-j\rvert^2}{N^2}\right)}$	40. Inverse Difference Normalized	$\sum_{i=1}^{N_g}\sum_{j=1}^{N_g}\frac{\mathbf{P}(i,j)}{1+\left(\frac{\lvert i-j\rvert}{N}\right)}$
41. Inverse variance	$\sum_{i=1}^{N_g}\sum_{j=1}^{N_g}\frac{\mathbf{P}(i,j)}{\lvert i-j\rvert^2}, i\neq j$	42. Maximum Probability	$\max(\mathbf{P}(i,j))$
43. Sum average	$\sum_{i=2}^{2N_g}[i\mathbf{P}_{x+y}(i)]$	44. Sum entropy	$-\sum_{i=2}^{2N_g}\mathbf{P}_{x+y}(i)log_2[\mathbf{P}_{x+y}(i)]$
45. Variance	$\sum_{i=1}^{N_g}\sum_{j=1}^{N_g}(i-\mu)^2\mathbf{P}(\mathbf{i},\mathbf{j})$		
**Textural features (gray-level run-length matrix based features)** $p(i,j\mid\theta)$, (*i,j*)th entry in the given run-length matrix *p* for a direction *θ*; N_g, number of discrete intensity levels in the image; N_r, number of different run lengths			
46. Short Run Emphasis	$\frac{\sum_{i=1}^{N_g}\sum_{j=1}^{N_r}\left[\frac{p(i,j\mid\theta)}{j^2}\right]}{\sum_{i=1}^{N_g}\sum_{j=1}^{N_r}p(i,j\mid\theta)}$	47. Long Run Emphasis	$\frac{\sum_{i=1}^{N_g}\sum_{j=1}^{N_r}j^2p(i,j\mid\theta)}{\sum_{i=1}^{N_g}\sum_{j=1}^{N_r}p(i,j\mid\theta)}$
48. Gray Level Non-Uniformity	$\frac{\sum_{i=1}^{N_g}\left[\sum_{j=1}^{N_r}p(i,j\mid\theta)\right]^2}{\sum_{i=1}^{N_g}\sum_{j=1}^{N_r}p(i,j\mid\theta)}$	49. Run Length Non-Uniformity	$\frac{\sum_{j=1}^{N_r}\left[\sum_{i=1}^{N_g}p(i,j\mid\theta)\right]^2}{\sum_{i=1}^{N_g}\sum_{j=1}^{N_r}p(i,j\mid\theta)}$
50. Run Percentage	$\sum_{i=1}^{N_g}\sum_{j=1}^{N_r}\frac{p(i,j\mid\theta)}{N_p}$	51. Low Gray Level Run Emphasis	$\frac{\sum_{i=1}^{N_g}\sum_{j=1}^{N_r}\left[\frac{p(i,j\mid\theta)}{i^2}\right]}{\sum_{i=1}^{N_g}\sum_{j=1}^{N_r}p(i,j\mid\theta)}$
52. High Gray Level Run Emphasis	$\frac{\sum_{i=1}^{N_g}\sum_{j=1}^{N_r}i^2p(i,j\mid\theta)}{\sum_{i=1}^{N_g}\sum_{j=1}^{N_r}p(i,j\mid\theta)}$	53. Short Run Low Gray Level Emphasis	$\frac{\sum_{i=1}^{N_g}\sum_{j=1}^{N_r}\left[\frac{p(i,j\mid\theta)}{i^2j^2}\right]}{\sum_{i=1}^{N_g}\sum_{j=1}^{N_r}p(i,j\mid\theta)}$
54. Short Run High Gray Level Emphasis	$\frac{\sum_{i=1}^{N_g}\sum_{j=1}^{N_r}\left[\frac{p(i,j\mid\theta)i^2}{j^2}\right]}{\sum_{i=1}^{N_g}\sum_{j=1}^{N_r}p(i,j\mid\theta)}$	55. Long Run Low Gray Level Emphasis	$\frac{\sum_{i=1}^{N_g}\sum_{j=1}^{N_r}\left[\frac{p(i,j\mid\theta)j^2}{i^2}\right]}{\sum_{i=1}^{N_g}\sum_{j=1}^{N_r}p(i,j\mid\theta)}$
56. Long Run High Gray Level Emphasis	$\frac{\sum_{i=1}^{N_g}\sum_{j=1}^{N_r}i^2j^2p(i,j\mid\theta)}{\sum_{i=1}^{N_g}\sum_{j=1}^{N_r}p(i,j\mid\theta)}$		
**Textural features (local binary pattern based features)** **X** denotes the vector of local binary pattern with *N* voxels in the tumor ROIs. The local binary pattern was estimated based on the relations of center pixel with 8 neighbors; $\overline{\mathrm{X}}$, the mean of **X**; **P**, the first-order histogram with N_l discrete intensity levels. Equations #1 to 16 (first order statistics) were then applied to yield 16 local binary pattern based features.			

Supplementary Table S3. List for the calculation of vasculature. [1]

Categories	Vasculature radiomics
1-5. Statistics of torsion per branch	Mean, standard deviation (std), maximum (max), skewness (skew), and kurtosis (kurt) of torsion across all branches
6-10. Statistics of curvature standard deviation per branch	Mean, std, max, skew, kurt of the standard deviation of curvature measured along each branch
11-15. Statistics of mean curvature per branch	Mean, std, max, skew, kurt of the average curvature measured along each branch
16-20. Statistics of maximum curvature per branch	Mean, std, max, skew, kurt of the maximum curvature measured along each branch
21-25. Statistics of curvature skewness per branch	Mean, std, max, skew, kurt of the skewness of curvature measured along each branch
26-30. Statistics of curvature kurtosis per branch	Mean, std, max, skew, kurt of the kurtosis of curvature measured along each branch
31-35. Statistics of global vascular curvature	Mean, std, max, skew, kurt of the curvature measured across all branches combined
36-45. Histogram of global vascular curvature	10-bin histogram of the curvature measured across all points of the vessel volume
46-55. Histogram of torsion	10-bin histogram of the torsion measured across all branches combined
56-58. Total vessel volume	Vessel volume (56), vessel volume normalized to the total size of the 3D region of interest (57), vessel volume normalized to the volume of the tumor (58).
59. Total vessel length	Total length of vessels within the region of interest
60-61. Tumor feeding branches	Number (60) and percentage (61) of vessel branches that enter the tumor volume from the surrounding tumor environment.
62-66. Statistics of vessel orientation along XY projection image	Mean, std, max, skew, kurt of the kurtosis of of local vessel orientations computed across XY vessel map
67-71. Statistics of vessel orientation along the XZ projection image	Mean, med, std, skew, kurt of local vessel orientations computed across XZ vessel map
72-76. Statistics of vessel orientation along the YZ projection image	Mean, std, max, skew, kurt of local vessel orientations computed across XZ vessel map
77-81. Statistics of vessel orientation along the rotation-elevation projection image	Mean, std, max, skew, kurt of local vessel orientations computed across vessel map of rotation and elevation with respect to the tumor
82-86. Statistics of vessel orientation along the distance-rotation projection image	Mean, std, max, skew, kurt of local vessel orientations computed across vessel map of distance and rotation with respect to the tumor
87-91. Statistics of vessel orientation along the distance-elevation projection image	Mean, std, max, skew, kurt of local vessel orientations computed across vessel map of distance and elevation with respect to the tumor

Supplementary Table S4. Results of grid search of hyper-parameters for DeepSurv models.

Number of hidden layers		
**Value**	**Average C-index**	**Average time cost (second)**
2	0.70	438
3	0.72	545
**4 (Selected)**	0.78	711
5	0.70	710
6	0.75	766
**Number of nodes in each hidden layer**		
**Value**	**Average C-index**	**Average time cost (second)**
4	0.70	489
8	0.74	530
16	0.71	563
**20 (Selected)**	0.77	555
24	0.76	514
32	0.75	603
64	0.76	648
**Initial learning rate**		
**Value**	**Average C-index**	**Average time cost (second)**
0.001	0.76	682
0.005	0.76	641
0.01	0.76	547
**0.05 (Selected)**	0.77	610
0.1	0.75	728
**Learning rate decay**		
**Value**	**Average C-index**	**Average time cost (second)**
**0.01 (Selected)**	0.83	449
0.001	0.72	652
0.0001	0.72	938
**Dropout rate**		
**Value**	**Average C-index**	**Average time cost (second)**
0.1	0.78	670
0.2	0.77	548
**0.4 (Selected)**	0.80	630

The hyper-parameters, including the momentum of 0.9, the epoch of 2000, the optimizer of Adam and the regularization of L2, are fixed. Bold: Selected hyper-parameters.

Supplementary Table S5. Selected features for the DeepSurv model training.

Feature Name	Feature Type or Filtering	Hazard ratio
**Clinical features**		
Patient age	-	1.25
Total protein	-	1.13
Mean corpuscular volume	-	1.22
**Intratumoral radiomics**		
Energy - Histogram	None, LLL, LHL, LHH, HLL, HHL, HHH	1.28-1.51
Kurtosis - Histogram	LLH	1.33
Maximum - Histogram	HHL	1.28
Surface area - Geometric	None	1.22
Volume - Geometric	None	1.48
Difference entropy - GLCM	LHL	1.33
Entropy - GLCM	LLH, LHL	1.28-1.32
Long run emphasis - GLRLM	None, LLH, LHH, HLH, HHL,HHH	1.34-1.43
Run length nonuniformity - GLRLM	LHH, HLH, HHL, HHH	1.40-1.47
Long run gray level emphasis - GLRLM	None, LLL, LLH, LHL, HLL, HHL	1.33-1.70
**Peritumoral-vasculature radiomics**		
Mean of torsion per branch	Morphology	1.37
Skewness of torsion per branch	Morphology	1.27
Bin 2, bin 3, bin 4 and bin 5 of global vascular curvature	Morphology	1.22-1.83

GLCM: gray-level co-occurrence matrix. **GLRLM**: gray-level run length matrix. In the column of wavelet filtering, L represents a low-pass filter, and H represents a high-pass filter. The combination of L and H letters stands for the filter type applied to the three image axes in order.

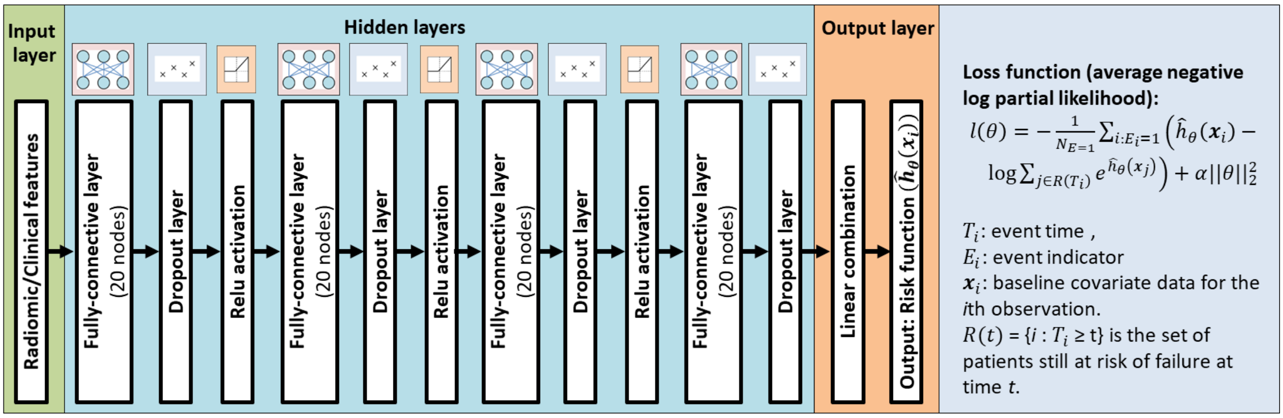

Supplementary Figure S1. The architecture of the implemented DeepSurv model. The DeepSurv models utilize radiomic (intratumoral and peritumoral vasculature) and clinical features as inputs. The inputs are processed through hidden layers with assigned weights. Each fully connected layer consists of 20 nodes and includes a 40% dropout. The training objective involves minimizing the average negative log partial likelihood.

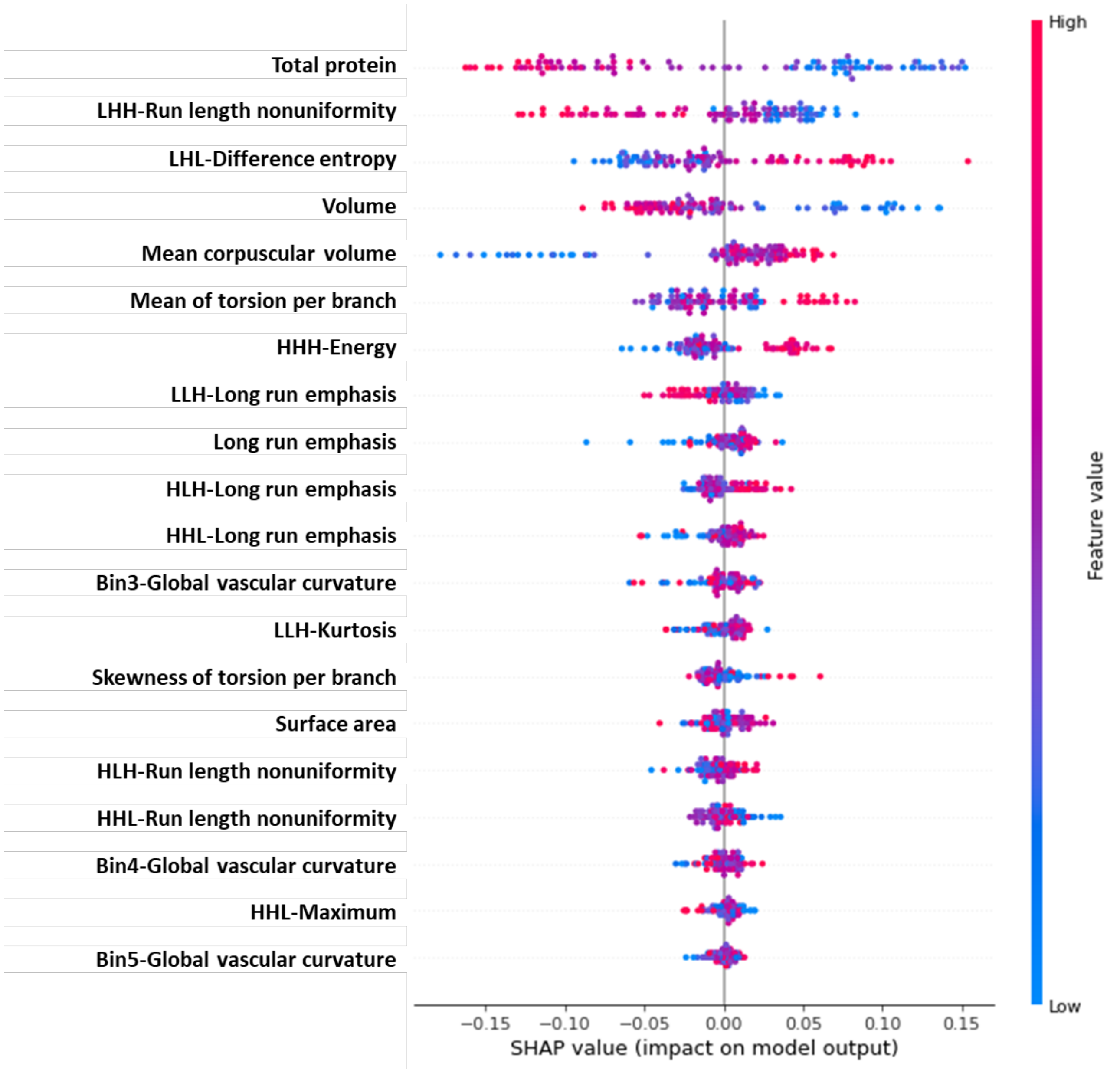

Supplementary Figure S2: Visualization of feature importance using SHAP analysis. This figure is a visualization of SHAP values, depicting the impact of various features on a model's output. SHAP values represent the contribution of each feature to the model's prediction. Each dot on the graph corresponds to an individual data sample, with its horizontal placement indicating the magnitude of the SHAP value, and its vertical placement corresponding to different features. Positive values (to the right) suggest a feature that positively influences the model's prediction, while negative values (to the left) indicate a negative influence. The color gradient from low (blue) to high (red) signifies the magnitude of the feature value itself. For example, a high total protein value positively affects the model's output, reflected by more points further from the zero point (center line).

Reference

1. Braman, N., et al., *Novel Radiomic Measurements of Tumor-Associated Vasculature Morphology on Clinical Imaging as a Biomarker of Treatment Response in Multiple Cancers.* Clinical Cancer Research, 2022. **28**(20): p. 4410-4424.
